# Super-resolution microscopy reveals glioma cell footprints and exosome deposits

**DOI:** 10.1080/19336918.2025.2534759

**Published:** 2025-07-24

**Authors:** Stefania Petrini, Frédéric Eghiaian, Valentina Apollonio, Giulia Pericoli, Maria Vinci

**Affiliations:** a Confocal Microscopy Core Facility, Research Center, Bambino Gesù Children’s Hospital, IRCCS, Rome, Italy; b Abberior Instruments GmbH, Göttingen, Germany; c Onco-Haematology and Pharmaceutical GMP Facility Research Area, Bambino Gesù Children’s Hospital, IRCCS, Rome, Italy

**Keywords:** CD63, exosomes, glioma, microscopy, STED

## Abstract

Gliomas are aggressive brain tumors whose infiltrative growth is mediated by intercellular crosstalk. Exosomes, small extracellular vesicles, play a key role in cell–cell communication but are difficult to visualize using conventional microscopy. Performing immunostaining for CD63, a known exosome marker, and using STED microscopy, we demonstrate exosome secretion in primary glioma cells. Applying mathematical deconvolution, we enhance the contrast and resolution for in-depth analysis of STED images. We identify CD63-positive cellular footprints and exosome deposits in the extracellular space. Quantitative analysis shows CD63-positive exosomes ranging 36.55-157.06 nm in size. CD63/actin co-staining demonstrates different actin polymerization states associated with exosomes. In conclusion, STED microscopy coupled with immunostaining allows exosome primary characterization at the single-vesicle level in the cellular spatial context.

## Introduction

High-grade gliomas are tumors of the central nervous system (CNS) [1,2] with disseminative growth, for which is difficult to control tumor progression and to develop effective therapies. Recently, it has been shown that the invasiveness of these tumors is sustained by the formation of functional networks between the tumor cells and between the tumor and the microenvironment. Gliomas form cell networks between heterogeneous tumor cell subpopulations that via functional cooperative mechanisms acquire a higher migratory and invasive capacity than the tumor cell subpopulations taken individually [3–5]. Then, within the microenvironment, neuron–glioma interactions are key in promoting tumor growth and glioma cell dissemination by the establishment of electrical circuits and secretion of active stimuli [6–14].

Glioma cells can use specialized structures called tumor microtubes (TMs) and gap junctions through which they invade and disseminate into the brain and interconnect with each other building a tumor cell network [6,9,15]. In addition to that, cell–cell communication requires the transmission of chemical signals from a sending cell to a receiving cell to coordinate several cell functions. This may occur through indirect cell–cell communication, such as paracrine and autocrine signaling, and by direct cell–cell contact, such as juxtacrine signaling [16].

In this context, extracellular vesicles (EVs) are cell-derived membrane-bound vesicles that provide paracrine, autocrine, and endocrine cell signaling, with an important role in cell differentiation, tissue development, homeostasis, and cancer development [17–19]. Based on their size, specific markers, and biogenesis, EVs are classified into four groups: exosomes, microvesicles, large EVs, and migrasomes [20–23]. Exosomes are the smallest EVs, with diameters between 40 and 160 nm [24]. Exosomes are secreted by cells and contain different biological molecules, such as nucleic acids, proteins, and lipids, reflecting the molecular signature of the cells of origin [24,25]. They mediate cell–cell communication and in tumour context, exosomes produced by cancer cells and by the cells of the tumour microenvironment contribute to tumour growth, metastasis, and therapy resistance [26]. In particular, exosomes have been shown to promote different aspects of cancer cell dissemination, mediating cell motility, cell adhesion, cellular polarization, and extracellular matrix remodeling [4,27–29]. We have recently shown that heterogeneous subpopulations of pediatric high-grade glioma (pHGG) cells secrete exosomes carrying different miRNAs and proteins and that the inhibition of exosome biogenesis results in reduced cell motility and the modulation of exosomal miRNA target gene expression. We showed that exosomes play a role in pHGG inter-clonal interactions and might play a key role in the dissemination of these tumors [4].

For the characterization of the EVs, several complementary techniques are recommended, as reported in the latest minimal information for studies of extracellular vesicles 2023 guidelines [30]. The number of EVs can be analyzed by light scattering technologies, such as nanoparticle tracking analysis (NTA), flow cytometry, or various colorimetric assays, such as Bradford or bicinchonic acid (BCA), to quantify the total protein amount. The other two aspects that are important for vesicle analysis are the specific molecular markers of EVs and the structure and diameter of EVs. In most cases, specific proteins expressed on the EVs are used as molecular markers and analyzed by western blotting. The structure and diameters are standardly examined using imaging techniques such as scanning electron microscopy, transmission electron microscopy, fluorescence microscopy and/or by flow cytometry. Although these techniques have different resolutions, they allow analysis of EVs smaller than 200 nm. Conventional fluorescence microscopy has been the most widely used technique for studying and analyzing the biogenesis and internalization of EVs; however, its detection limitations have not allowed in-depth information on cell-EV interactions and their subcellular localization owing the diffraction limit of light, that is 200 nm in lateral resolution. In fact, the 3D image of a single point light source seen through the microscope, the so-called point spread function (PSF), is strongly dependent on known optical parameters and wavelength of the illumination, and it is the result of the acquired image convolution with the blurring contribution. The application of iterative algorithms that use a PSF to correct the optical aberrations that are specific to a given microscope, increases the signal-to-noise ratio (S/N) and improves the contrast, allowing the recovery of an image degraded by the convolution, operating an image restoration process called deconvolution. The use of specific deconvolution algorithms for images acquired in conventional microscopy has become an important computational tool for increasing the quality and resolution of blurred 2D and 3D images, allowing a more precise quantitative dimensional analysis (*e.g*. shape and size of nanoscopic structures). Although these mathematical deconvolution processes have allowed to achieve a resolution improvement of 120–140 nm, which is insufficient to resolve individual vesicles. Great progress in the characterization and biology of EVs has been achieved with the advancements of super-resolution microscopy (SRM) techniques that allow investigating at nanoscale resolution various aspects of EV biology, such as the packaging of cargo and the interaction between EVs and cellular components [31,32]. SRM techniques include single-molecule localization microscopy (SMLM) technologies, represented by photoactivated localization microscopy (PALM), stochastic optical reconstruction microscopy (STORM), DNA-points accumulation for imaging in topography (DNA-PAINT), which are able to provide a lateral resolution lower than 20 nm, and the minimal photon fluxes (MINFLUX) technique allowing to reach 1–3 nm of resolution [33]. Both SMLM and MINFLUX rely on photo-switchable fluorophores and on the reconstruction of a series of resolution images recorded at different time frames and have been mostly used in EV research [32]. In addition, another SRM category providing spatial resolution below the light optical diffraction limit is represented by stimulated emission depletion (STED) microscopy [34] which is well suited to characterize the size and distribution of fluorescently labeled EVs and small vesicles by selectively deactivating photostable fluorophores [35]. In STED microscopy, the excitation beam focused on the sample is coaligned with a second red-shifted laser beam called the STED beam, which is arranged in a doughnut shape thus allowing the depletion of the fluorophores located at the periphery of the doughnut [33,34,36,37]. In this way, the central focal spot remains unaffected by this de-excitation, and it is super-resolved with a dimension of 45 nm, well below the diffraction limit [35].

In this study, we performed immunofluorescent staining for CD63, a known EV marker [38,39], on a primary glioma patient-derived cell line and we used a STED microscopy platform to demonstrate EV secretion directly in cultured cells. Mathematical deconvolution tools were applied to STED images for accurate characterization of nanovesicle size and distribution, proving to be excellent complements to STED imaging.

## Results and discussion

The observation of EVs at the smallest scale is fundamental in oncology research to better understand the cell signaling and cell–cell communication processes underlying cancer development and dissemination.

**Figure 1.**
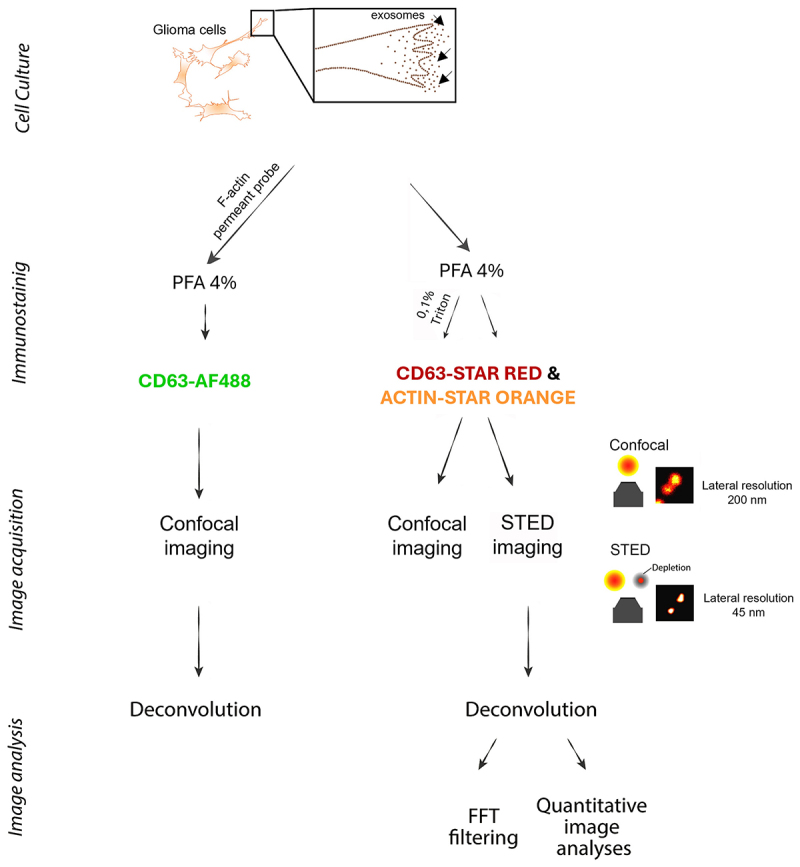
Schematic illustration showing the workflow and experimental setup used for the study.

In our study, differently from previous reports where EVs were collected from cell culture media and then either seeded on petri dishes for direct imaging or added to other cells for studying cellular up-take [31], we used a combination of microscopy and immunolabeling approach to analyze the EVs secreted by primary cells directly in culture. For this, we compared conventional confocal microscopy to STED microscopy, and we used a primary patient-derived glioma cell line, as a cell model to investigate the applicability on fixed cells of a primary antibody against CD63, a common exosome marker, to detect secreted exosomes in glioma cells cultured on an ECM substrate such as laminin. In co-staining with CD63, we used an antibody against actin to also label the cytoskeleton and thus, facilitate the detection of CD63-positive exosomes relative to the cell body (Figure 1 and Supplementary Table S1). We performed an indirect immunofluorescence method to improve the labeling density, making use of secondary antibodies tagged with STAR Red and STAR Orange fluorophores, known for their photostability, brightness, and low background [40].

Previous confocal microscopy studies have already demonstrated the subcellular and extracellular distribution of vesicles in the form of aggregates or discrete domains of the plasma membrane [41–43], but the same studies have also shown the strong limitation of this system in the identification and analysis at the single-vesicle level. SRM techniques, for example, STORM and PALM have been used in EV qualitative and quantitative characterization analyses [31,44,45] providing better spatial resolution at the nanometer scale (≤20 nm) but are time-consuming since a large number of collected images during the repetitive imaging procedure have to be performed. These SRM approaches, as also happens in 2D STED, depend on high photon intensities and are not suited for prolonged EV live imaging, whereas they find their best application on fixed samples. Among the SRM techniques, STED microscopy has provided an important contribution to the identification of EVs at the single-vesicle level [46–48] and is well used for vesicle size determination in fixed cellular samples, as for our experimental set-up.

**Figure 2.**
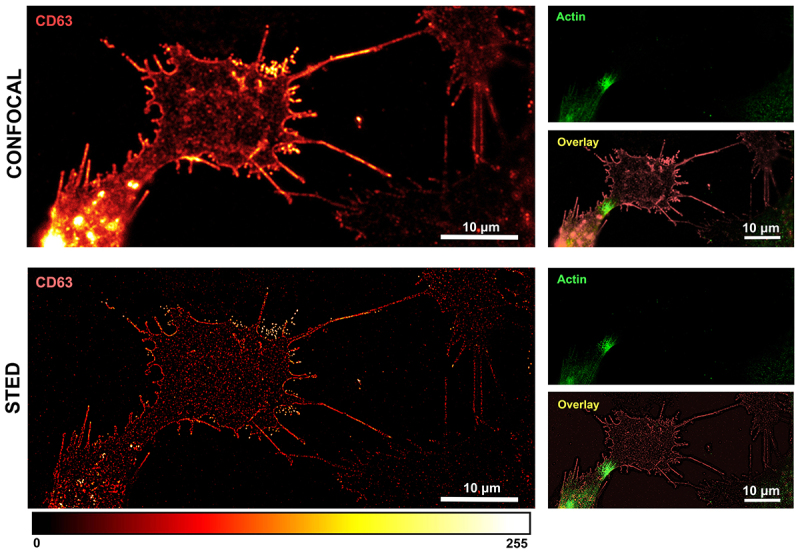
Comparison of confocal and STED microscopy. Representative images of OPBG-GBM001 glioma cells fixed and immunostained with anti-CD63 (pseudo-colored in red) and anti-actin (pseudo-colored in green) antibodies revealed by STAR Red and STAR Orange-conjugated secondary antibodies, respectively. Confocal and STED images were acquired with a single microscopy platform that works with both modalities and the same field of view was captured. Confocal imaging (upper panel) revealed the presence of clusters of CD63-positive dots, close to the plasma membrane. In the deconvolved STED image (lower panel), single CD63-positive dots appear individually resolved at the nanoscale level and the plasmalemma shows a discontinuous, dotted CD63-positive signal. Polymerized actin filaments (right) are not detected in the enlarged, featured area of the cell. The fluorescence intensity is shown by the ‘Red Hot’ LUT scale of the ImageJ software.

In this study, we used a nanoscopy platform that works in both confocal and STED modalities. In confocal imaging (Figure 2, upper panel), we observed CD63 positive staining which appeared as glioma cellular footprint-like structures. These structures were subsequently better resolved by acquiring the same field of view with STED microscopy. After deconvolution by Huygens software using a classic maximum likelihood estimation (CMLE) algorithm, STED images were further improved via fast Fourier transformation (FFT) [49] to reduce the out-of-focus noise (Figure 1). The STED images enabled us to visualize the staining with CD63-positive dots precisely demarcating the cell membrane also with filopodia and finger-like projections (Figure 2, lower panel). CD63-positive dots were also present on the cellular footprint [50,51] and interestingly, brighter dots were seen in the extracellular space. The staining appears specific, as suggested by the images of the negative controls incubated with the secondary antibodies in the absence of primary antibodies (Suplementary Figure S1). CD63 is a tetraspanin protein from the Trans Golgi Network (TGN) that is either shuttled directly on the plasma membrane or the intraluminal vesicles (ILVs) and the multivesicular bodies (MVBs). When these fuse with the plasma membrane, they release exosomes enriched in CD63 [39]. In line with this, our data suggest that the brighter CD63 positive dots observed in the extracellular compartment are likely exosomes.

**Figure 3.**
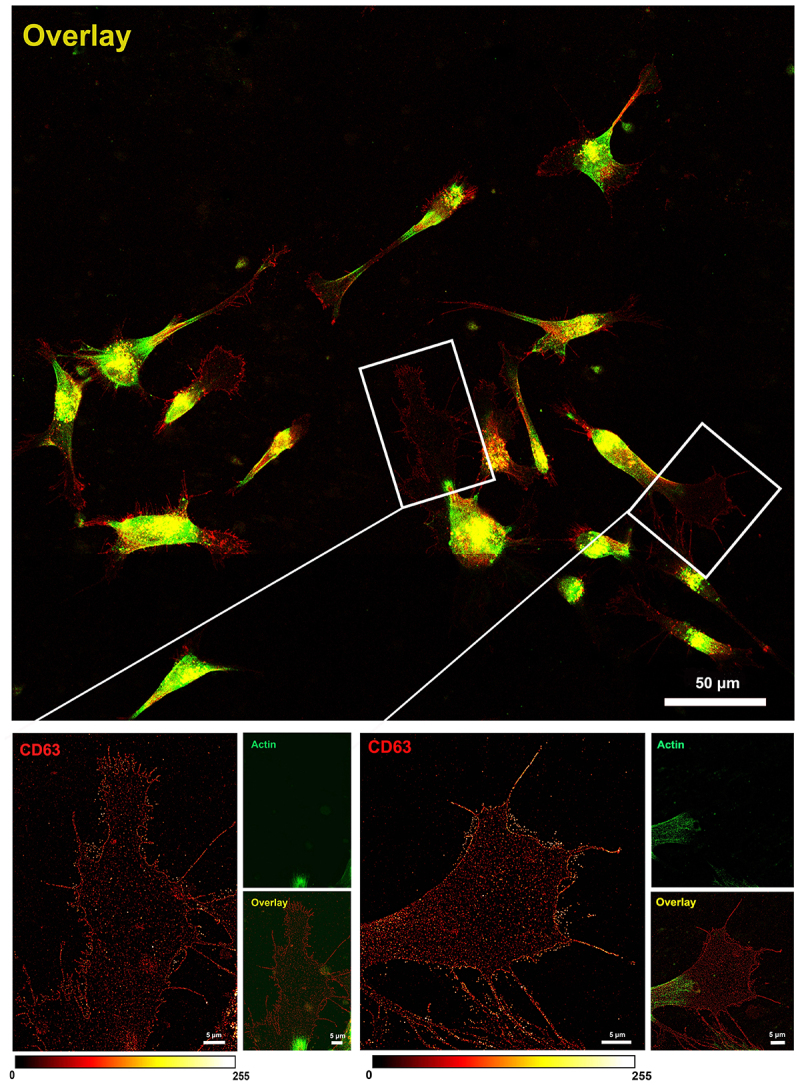
Comparison of confocal and STED microscopy. Representative images of OPBG-GBM001 glioma cells fixed and immunostained with anti-CD63 (pseudo-colored in red) and anti-actin (pseudo-colored in green) antibodies revealed by STAR Red and STAR Orange-conjugated secondary antibodies, respectively. Confocal and STED images were acquired with a single microscopy platform that works with both modalities and the same field of view was captured. Confocal imaging (upper panel) revealed the presence of clusters of CD63-positive dots, close to the plasma membrane. In the deconvolved STED image (lower panel), single CD63-positive dots appear individually resolved at the nanoscale level and the plasmalemma shows a discontinuous, dotted CD63-positive signal. Polymerized actin filaments (right) are not detected in the enlarged, featured area of the cell. The fluorescence intensity is shown by the ‘Red Hot’ LUT scale of the ImageJ software.

Of note, in both confocal and STED acquisition modalities, the signal of the polymerized actin was not detected on the cellular footprint-like structures (Figure 1, right panel). In fact, looking at STED images at lower magnification, it was evident that all the glioma cells examined in our experimental condition (cells adherent, fixed, and non-permeabilized) present CD63-positive/actin-negative cellular footprints. These structures display protrusions with different shapes and sizes depending on the cell movements and cell–cell interactions that naturally occurred before cell fixation (Figure 3, overlay image).

At higher power magnification, the CD63-positive glioma cellular footprints present as relatively large cell area characterized by punctuated CD63 staining on the cell membrane which, as displayed in the example featured in Figure 2, show finger-like projections and filopodia often reaching toward and/or connecting to neighbour cells (Figure 3, upper panel). Outside the cell membrane boundaries, we observed again several deposits of bright CD63-positive vesicles which are likely secreted exosomes (Figure 3, lower panel images).

To demonstrate that the secreted vesicles are indeed exosomes, we then super-resolved the individual vesicles by a combination of marker expression and size distribution analyses.

**Figure 4.**
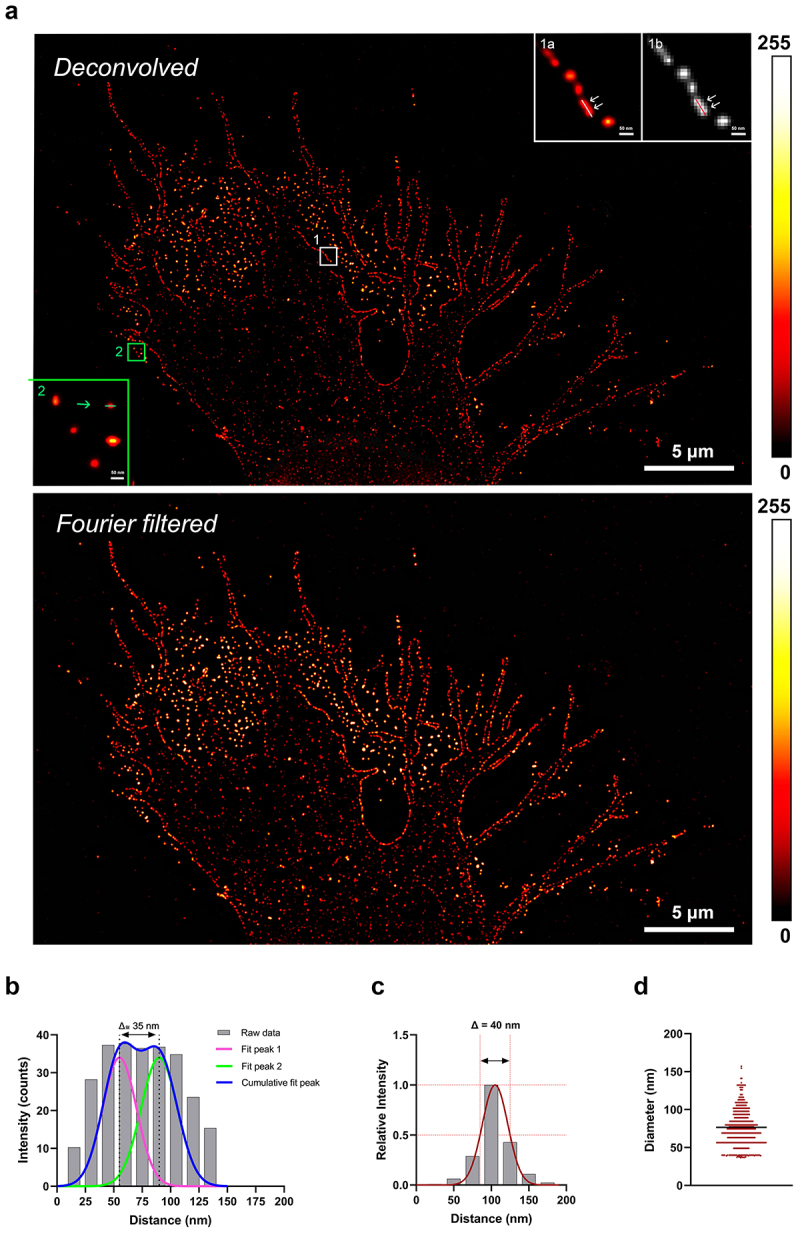
STED imaging of paraformaldehyde-fixed and permeabilized OPBG-GBM001 glioma cells. IF was performed with anti-CD63 (pseudo-colored in red) and anti-actin (pseudo-colored in green) primary antibodies revealed by STAR Red and STAR Orange-conjugated secondary antibodies, respectively. STED images were deconvolved, and then FFT filtered. In the large field of view (upper panel), glioma cells show CD63 and actin positive staining which in the distal area presents for both markers a dot-like pattern (inset, high magnification, lower panel). The inset on the high-power overlay image shows CD63-positive vesicles colocalizing with F-actin fibers (yellow dots), but not in the more distal cell regions where G-actin is detected (arrows). Scale bar: ‘Red Hot’ LUT intensity scale.

For the analysis, we took advantage of Huygens deconvolution software for raw STED images and obtained deconvolved STED (dSTED) images (Figure 4a). These were further improved by subtracting the non-homogeneous out-of-focus noise via FFT for better visualization (Figure 4a, Supplementary Figure S2a). When comparing the deconvolved and Fourier-filtered STED image, we obtained a fine resolution at the level of single CD63-positive dots in the dSTED image, whereas in the FFT-filtered image the signal-to-noise (S/N) ratio was improved.

From the comparison between the two different microscopy techniques used in this study, confocal and STED, and between dSTED and FFT-filtered dSTED images, the greatest values of S/N and signal-to-background (S/B) ratios were observed in the latter condition (Supplementary Table 1). However, the application of a filter in the frequency domain, such as that of the Fourier transformation, can determine the execution of various smoothing operations, which in our images translates into a variation (*i.e*. slight enlargement) of the exosome sizes and a decreased lateral resolution (Supplementary Figure S2B and C). Taking into account this aspect, we considered more correct and precise the dSTED images to carry out the quantitative analysis of the diameter of the CD63-positive dots. In our experimental conditions, considering the instrument acquisition settings and the deconvolution approach used in reconstructing super-resolution STED images [52] we measured the Full-Width Half Maximum (FWHM, i.e. the width of the peak at half of its maximum value) of the intensity plot profiles of lines drawn between two very close, although distinct, CD63 fluorescent dots, and we achieved a 35 nm lateral resolution (Figure 4a, white line in the inset 1a, red line in the inset 1b; Figure 4b). As for the size of the measured dots, the distribution of FWHM values ranged from a minimum of 36,55 nm (~40 nm), like the one represented in the line profile (Figure 4c, green line in the inset 2), to a maximum of approximately 157,06 nm (~160 nm). The average value found by analyzing 450 EVs secreted by 3 glioma cells is equal to 76,55 ± 24,99 nm (Figure 4d). Taking into account a possible bias introduced by the indirect immunofluorescence in sizing (that may lead to a maximum increase of 30 nm in the size of the observed fluorescent object [53], the minimum and maximum diameter we measured (~40–160 nm), fall in within the size range of exosomes thus demonstrating that the CD63-positive dots found in the extracellular compartment of the glioma cells are indeed exosomes (Figure 4d).

**Figure 5.**
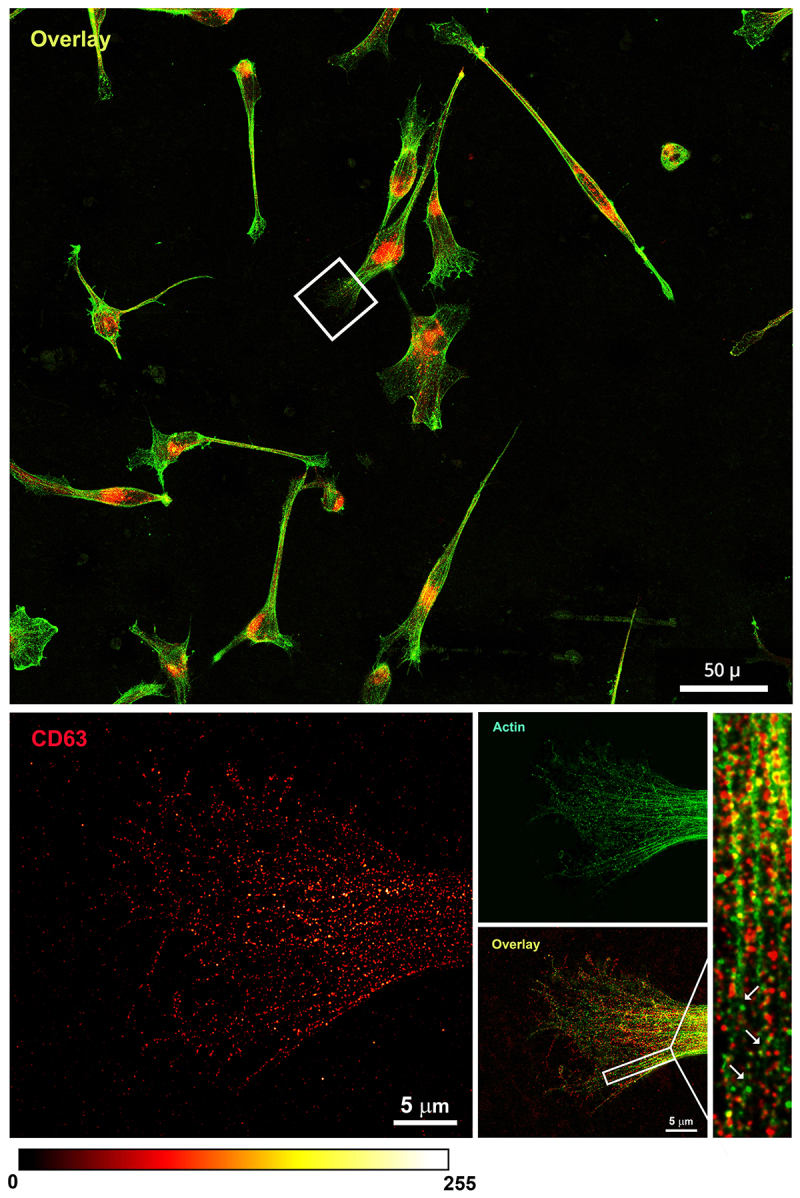
STED imaging of paraformaldehyde-fixed and permeabilized OPBG-GBM001 glioma cells. IF was performed with anti-CD63 (pseudo-colored in red) and anti-actin (pseudo-colored in green) primary antibodies revealed by STAR Red and STAR Orange-conjugated secondary antibodies, respectively. STED images were deconvolved, and then FFT filtered. In the large field of view (upper panel), glioma cells show CD63 and actin positive staining which in the distal area presents for both markers a dot-like pattern (inset, high magnification, lower panel). The inset on the high-power overlay image shows CD63-positive vesicles colocalizing with F-actin fibers (yellow dots), but not in the more distal cell regions where G-actin is detected (arrows). Scale bar: ‘Red Hot’ LUT intensity scale.

Our data demonstrate that super-resolution STED technology coupled with the use of a specific marker such as CD63 [39] can be used to precisely detect and measure individual EVs secreted by primary patient-derived glioma cells. Our experimental set-up is ideal for the study of secreted exosomes with great advantage compared to other more commonly used approaches [30]: we can visualize at the nanoscale level and characterize in depth the EVs released in the extracellular space without the need to perturb the cells from their specific culture condition. This is particularly advantageous for delicate primary cells, it enables performing the primary characterization of the exosomes in their spatial contexts, vesicles, and cells are less damaged compared to serial centrifugation or other analytical procedures, and it is particularly advantageous for conducting studies also with a relatively small number of cells and exosomes. It has to be noted, though, that in our study, we have potentially analyzed a subpopulation of exosomes, which adhered to the substrate, and which may be distinct from other exosomes secreted by the same parental cells, also released in the medium but washed away before cell fixation. Further studies will need to be performed to demonstrate this hypothesis, which could be explained by a heterogeneous expression of adhesion molecules on the exosome membrane. This would not be surprising and is in line with the heterogeneity we have already described in pediatric high-grade glioma cell lines and their exosomal cargo [4]. As shown in Figures 2 and 3, in the distal area of the glioma cell body it is possible to recognize cellular footprints identifiable by the CD63-positive staining on the plasma membrane and by the secreted EVs into the extracellular environment. Interestingly, on the same cell protrusions, the actin signal was weak/not detected. It is important to note, though, that in our experimental condition, the cells were not permeabilized prior to immunolabelling. To test the effect of the permeabilization on the visualization of the unique CD63 staining pattern, we performed co-immunofluorescence staining for CD63 and actin, but this time, cells were permeabilized. In this condition, the actin signal was detectable also in the cell protrusions, where we can appreciate the specific staining of F-actin co-localizing with CD63-positive vesicles as well as G-actin dot-like pattern in the more distal area of the cell protrusions (Figure 5). This data is in line with previous reports on the dynamics of the cytoskeleton rearrangements with an accumulation of G-actin at the leading edge during the process of cell migration [54–57].

To further confirm the polymerization and non-polymerization status of the actin in the cell protrusions, we performed staining in live cells using a specific permeant probe for F-actin filaments (SiR-Actin), and after that, cells were fixed and immunolabelled with anti-CD63 antibody. Taking into consideration the experimental differences between actin-labeled cells in permeabilized (Figure 5) and not permeabilized (Supplementary Figure S3) conditions, the actin staining pattern appears comparable with the absence of F-actin at the very distal area of the cell protrusions. In these regions, CD63-positive vesicles, showed a close association with the actin filaments (Figure 5) that function as tracks for their secretion, to then head toward the cell periphery where G-actin was undergoing polymerization.

Our findings confirm a close association between the vesicles secretion and the active polymerization dynamics of the actin cytoskeleton during cell migration.

Further, our results suggest that the unique CD63-positive cellular footprints, as well as the exosome secretion in the extracellular compartment, are best visualized when cells are not permeabilized and imaged using SRM techniques such as STED microscopy.

## Conclusion and future perspectives

In conclusion, by using STED microscopy, we show for the first time the formation of footprint-like structures rich in CD63-positive secreted exosomes in primary patient-derived high-grade glioma cells. The STED microscopy allowed the detection of the CD63-positive exosomes at the single-vesicle level in cultured cells while adherent to a laminin substrate, without the need for purifying exosomes from the conditioned medium. The glioma cells displayed numerous morphological patterns typical of highly migrating cells, which in their distal protrusions presented active dynamics of the actin cytoskeleton, CD63-positive traced plasma membrane, and numerous exosomes released in the extracellular space. In addition, the use of mathematical deconvolution tools contributed to increasing the contrast of raw STED images and achieving a 35 nm lateral resolution, providing a more accurate characterization of exosome distribution and dimensions. Future applications of this proof-of-concept study may include using a 3D STED platform equipped with adaptive illumination for lower light dose to reduce the sample phototoxicity in addition to the gas and temperature control chamber for live imaging. This, in conjunction with the use of reporter assay for CD63 and/or additional exosome-specific markers, would allow for live tracking of exosome secretion and for studying the dynamics of exosomes in the glioma intercellular communication during migration/invasion. STED technologies and other SRM technologies may aid in the finer definition of the multiple aspects of exosome biogenesis, release, and active role in cell–cell communication and cell motility in aggressive glioma cells, as well as other cancer cell types.

## Experimental section

### Cell cultures

The diffuse hemispheric glioma H3G34-mutant (DHG H3G34-mutant) patient-derived cell line, OPBG-GBM001, was established from cryopreserved tissue obtained from a patient at Ospedale Pediatrico Bambino Gesù. Patient tissue was collected following the rules of the Institutional Ethical Committee of the Bambino Gesù Children’s Hospital (Ethical Committee Approvals N°1680/2018). Written informed consent was obtained by patient’s legal guardians. The study was conducted according to the guidelines of the Declaration of Helsinki and approved by the Institutional Ethical Committee of the Bambino Gesù Children’s Hospital.

Cells were cultured as previously described [3]. Briefly, cells were grown on laminin (Merck) in tumor stem-cell media (TSM), composed by: 1:1 Neurobasal(−A) (Invitrogen), and DMEM: F12 (Invitrogen), supplemented with Antimycotic/Antibiotic, HEPES, NEAA, GlutamaX, Sodium Pyruvate (Invitrogen) and B27(−A) (Invitrogen), human bFGF (20 ng/mL), human EGF (20 ng/mL), human PDGF-AA (10 ng/mL) and PDGF-BB (10 ng/mL) (Peprotech), and heparin (2 ng/mL) (Stem Cell Technologies). The cell authenticity was verified using short tandem repeat (STR) DNA fingerprinting by Eurofins Genomics. Cells were tested and verified mycoplasma-free.

### Immunofluorescence

OPBG-GBM001 cells were seeded into plastic-chambered glass microscope slides (BD Falcon). Forty-eight hours after seeding, cells were fixed with 4% paraformaldehyde (PFA) (Bio-Optica) for 10 min followed or not by a permeabilization step with 0,1% Triton in PBS for 5 min. After incubation for 1 h at RT with a blocking solution (5% Bovine Serum Albumin (BSA, #10775835001, Roche, Basilea, Switzerland) in PBS, the following antibodies, mouse anti-CD63 (MX-49.129.5, Santa Cruz Biotechnology), and rabbit anti-Actin (Abcam) antibodies, were diluted 1:50 in IFF solution (1% BSA, 2% Fetal Bovine Serum (FCS) in PBS) overnight, at 4°C. Secondary goat immunoglobulins conjugated to the dyes STAR Red and STAR Orange (Abberior, Germany) were diluted 1:200 in IFF solution and incubated for 1 h at R.T. Negative controls were performed using PBS/BSA without the primary antibody with the addition of Hoechst as nuclear probe. All experiments were repeated twice. After three washes in PBS, slides were mounted using mount solid antifade mounting medium (Abberior), and #1.5 thickness coverslips.

In parallel experiments, the SiR-Actin permeant probe (SC001, Spirochrome, Cytoskeleton, Denver, CO) was added to the culture medium at final concentration of 1 mM and incubated for 1 h at 37°C; after fixation in 4% PFA for 10 min, washing in PBS, and blocking with 5% BSA in PBS for 1 h at RT, glioma cells were incubated with anti-CD63 antibody (overnight at 4°C) and secondary goat anti-mouse AlexaFluor 488-conjugated (Life technologies, 1 h, at RT) antibodies. Negative controls were performed using PBS/BSA without the primary antibody. All experiments were repeated twice. Samples were mounted with PBS/glycerol 60% and acquired on the Leica TCS-SP8X laser-scanning confocal microscope (Leica Microsystems, Mannheim, Germany).

### Confocal microscopy

Confocal microscopy was performed on a Leica TCS-SP8X laser-scanning confocal microscope (Leica Microsystems, Mannheim, Germany) equipped with tunable white light laser (WLL) source, 405 nm diode laser, 3 Internal Spectral Detector Channels (PMT), and 2 Internal Spectral Detector Channels (HyD) GaAsP. Sequential confocal images have been acquired using HC PL APO 63x/oil-immersion objectives (1.40 numerical aperture, NA, Leica Microsystems). Z-reconstructions of serial single optical sections have been acquired with a 1024 × 1024 format with a pixel resolution of 45 nm, scan speed of 200 Hz, and z-step size of µ0.3 m, using LAS X software (version 3.5). Contrast and resolution of confocal raw Z stacks frames were improved (up to ~140 nm of lateral resolution) by applying a deconvolution process (Hyvolution2 software, Huygens, Scientific Volume Imaging, SVI, The Netherlands, embedded in the Leica TCS-SP8X).

### Super-resolution STED microscopy

Glioma cells were immunostained using CD63 and Actin cross-adsorbed and revealed with specific goat secondary antibodies conjugated to STAR Red and STAR Orange dyes (Abberior GmbH, Germany), respectively. STED imaging was performed on a microscope platform (STEDYCON, Abberior Instruments GmbH, Germany) operating in both, confocal and STED modality, equipped with an upright Zeiss Axioimager Z.2 microscope and a pulsed STED laser with a depletion wavelength of 775 nm working at a repetition rate of 40 MHz, a pulse duration of 1 ns, using STEDYCON SmartControl software version 9. STAR RED was imaged with a pulsed source at a wavelength of 640 nm (detection range between 650 and 700 nm, gated detection between 1 and 7 ns), whereas STAR Orange was excited with a pulsed source at 561 nm (detection range between 575 and 625 nm, gated detection between 1 and 7 ns). The pixel size was 20 nm, and the pinhole was set to 1.1 Airy Units at 650 nm. For STED imaging, an oil-immersion objective lens was used (100x PlanApochromat oil-immersion objective, 1.46 NA, Zeiss, Germany). To improve resolution and contrast, an automatic deconvolution process (STED Deconvolution software, Huygens, SVI) using the inbuilt routines designed specifically for raw STED images, allowing to obtain a theoretical estimation of the STED PSF based on calculated values from the acquired image metadata [52]. The deconvolution process was completed by using an auto setting in Huygens. Detailed parameters were as follows: (1) background: automatic estimation, (2) estimate mode: lowest, (3) area radius: 0.7, (4) deconvolution algorithm: classic maximum likelihood estimation (CMLE), (5) maximum iteration: 27, (6) signal-to-noise ratio: 8, (7) quality threshold: 0.01, (8) iteration mode: optimized, (9) brick layout: auto.

Then deconvolved STED images were Fourier filtered [49] in order to perform image denoising, using the following steps: Process - > FFT (Fast Fourier Transforms) - > Bandpass Filter command tool: filter large structures down to 30 pixels; filter small structures up to 2 pixels, available through the ImageJ (*http://imagej.nih.gov/ij/*) open-source software. Representative 8 bit images were prepared in ImageJ using the ‘Red Hot’ Lookup Table (LUT) scale, then assembled in Photoshop software (Adobe Systems Inc., San Jose, CA, USA).

### Signal to noise measurements

The signal to noise (S/N) measurements were performed with ImageJ software for confocal, STED, deconvolved STED (dSTED), and FFT filtered dSTED (dSTED + FFT) images using freehand circular region to delimit the signal intensities of single exosomes (for CD63 staining) or regions where actin appears in the polymerized or depolymerized condition. Similarly, signal to background (S/B) ratio values were obtained in areas close to EVs (for CD63 staining) using freehand circular regions. About 15 counts, for each ROI, were performed in 3 different glioma cells.

### Exosomes measurements

Deconvolved STED (dSTED) images were processed using ImageJ open-source software in order to perform measurements of the diameter of exosome secreted by glioma cells and located in spatial proximity of the cell periphery. About 450 exosomes from 3 OPBG-GBM001 cells were measured, and the resolution plot and the intensity line profile of the exosome width was obtained using MetaMorph (Molecular Devices, San Jose, CA, USA) software. Quantitative results, reported as mean ± standard deviation (SD), were analyzed with GraphPad Prism software (Prism 8.0.2, GraphPad Software, San Diego, CA, USA) and Microsoft Excel (Microsoft, Redmond, Washington, DC, USA).

## Supplementary Material

Supplemental Material

## Data Availability

The data that support the findings of this study are available from the corresponding author, Maria Vinci, upon reasonable request.
