# Supplementary material for: Super-resolution microscopy reveals glioma cell footprints and exosome deposits
Source: Cell Adh Migr. 2025 Jul 24;19(1):2534759. doi: 10.1080/19336918.2025.2534759 (PMC12309551; doi:10.1080/19336918.2025.2534759)
Supplement: Supplemental Material [file KCAM_A_2534759_SM1646.docx]

Supplementary Figure 1


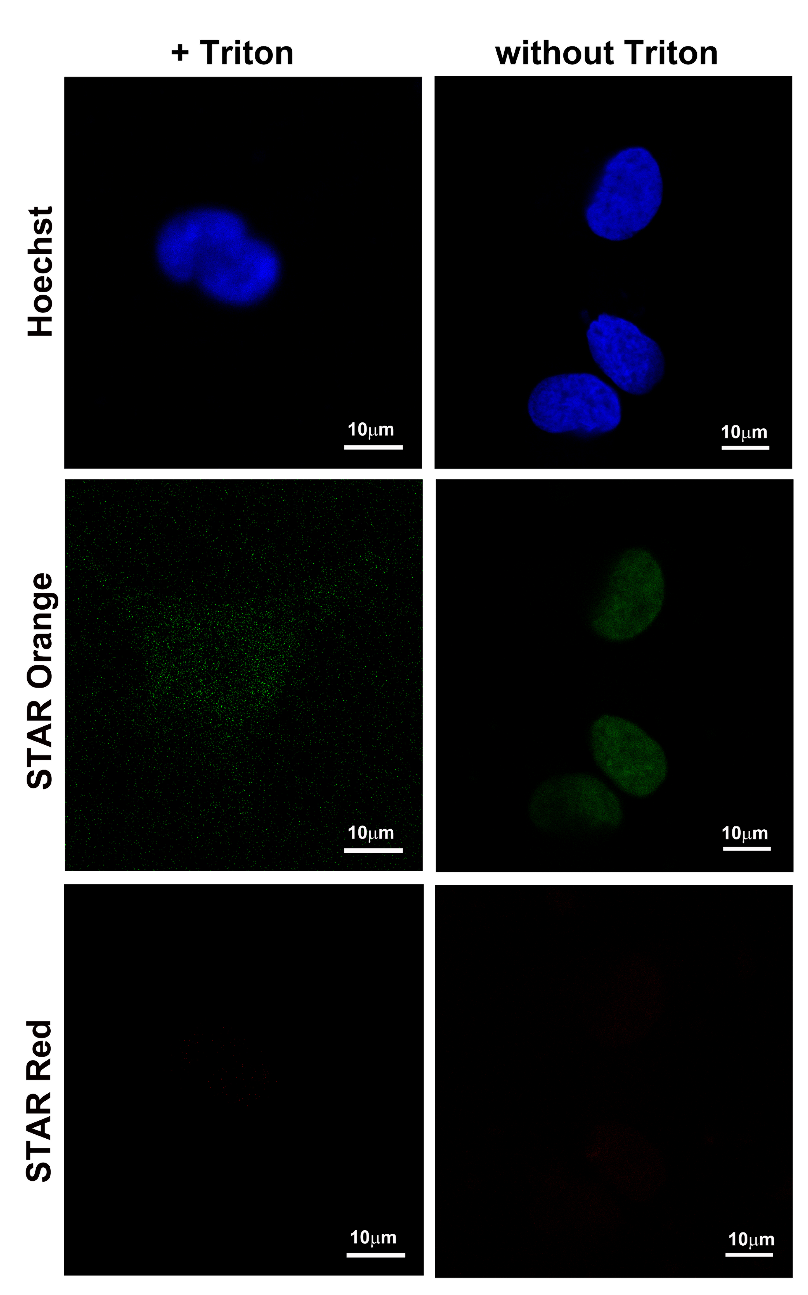


Supplementary Figure 1 alt text: Confocal and STED negative control images of permeabilized (+Triton) and non-permeabilized (without Triton) glioma cells stained with secondary antibodies only, showing no or low non-specific binding.

**Supplementary Figure 1** – Confocal (Hoechst channel) and STED (STAR Red and STAR Orange channels) images of negative control samples fixed in paraformaldehyde, in permeabilized (+Triton) and unpermeabilized (without Triton) conditions, incubated with the secondary fluorophore-conjugated antibodies, in the absence of the primary antibodies

Supplementary Figure 2


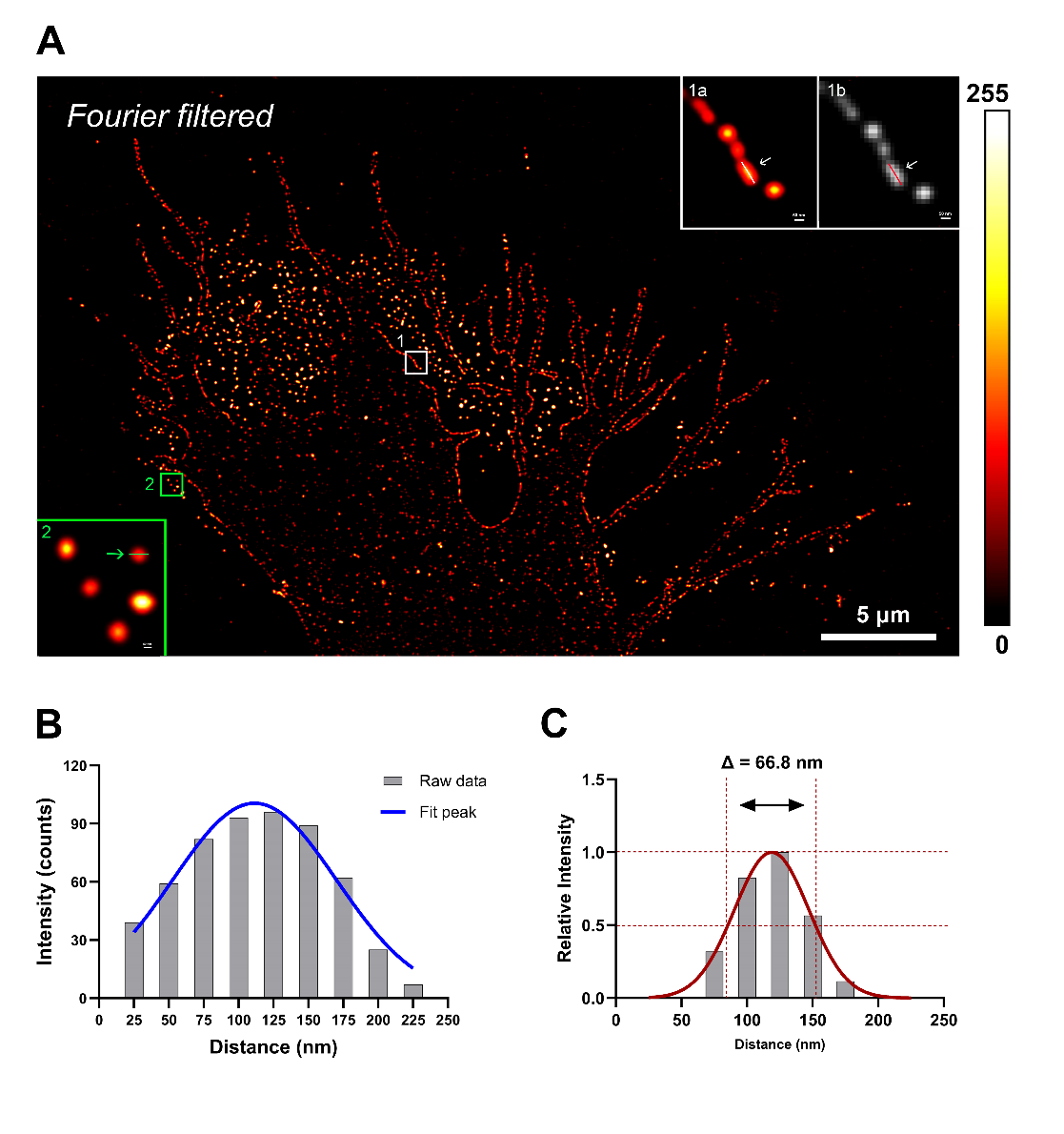


Supplementary Figure 2 alt text: Fourier filtered deconvolved STED image of CD63-labelled glioma cells. Magnified view of exosomes used to assess lateral resolution and diameter with corresponding plots.

Supplementary Figure 2 – (A) Representative deconvolved and Fourier filtered STED image of OPBG-GBM001 cells stained with anti-CD63 primary and STAR Red-conjugated secondary antibodies with the visualization of the lateral resolution achieved by examining the two exosomes (inset 1a, arrow) and their pixel intensity distribution (inset 1b, arrow) also examined in Figure 4A. (B) Cross-sectional spatial resolution plot along the white (inset 1a) and red (inset 1b) lines identifying a single exosome and not two as shown in Figure 4B. (C) Intensity line profile through the same exosome indicated in the inset 2 as in Figure 4A, showing the increase of its mean diameter in dSTED FFT-filtered image compared to the same analysis performed on dSTED image (Figure 4, A and C).

Supplementary Figure 3


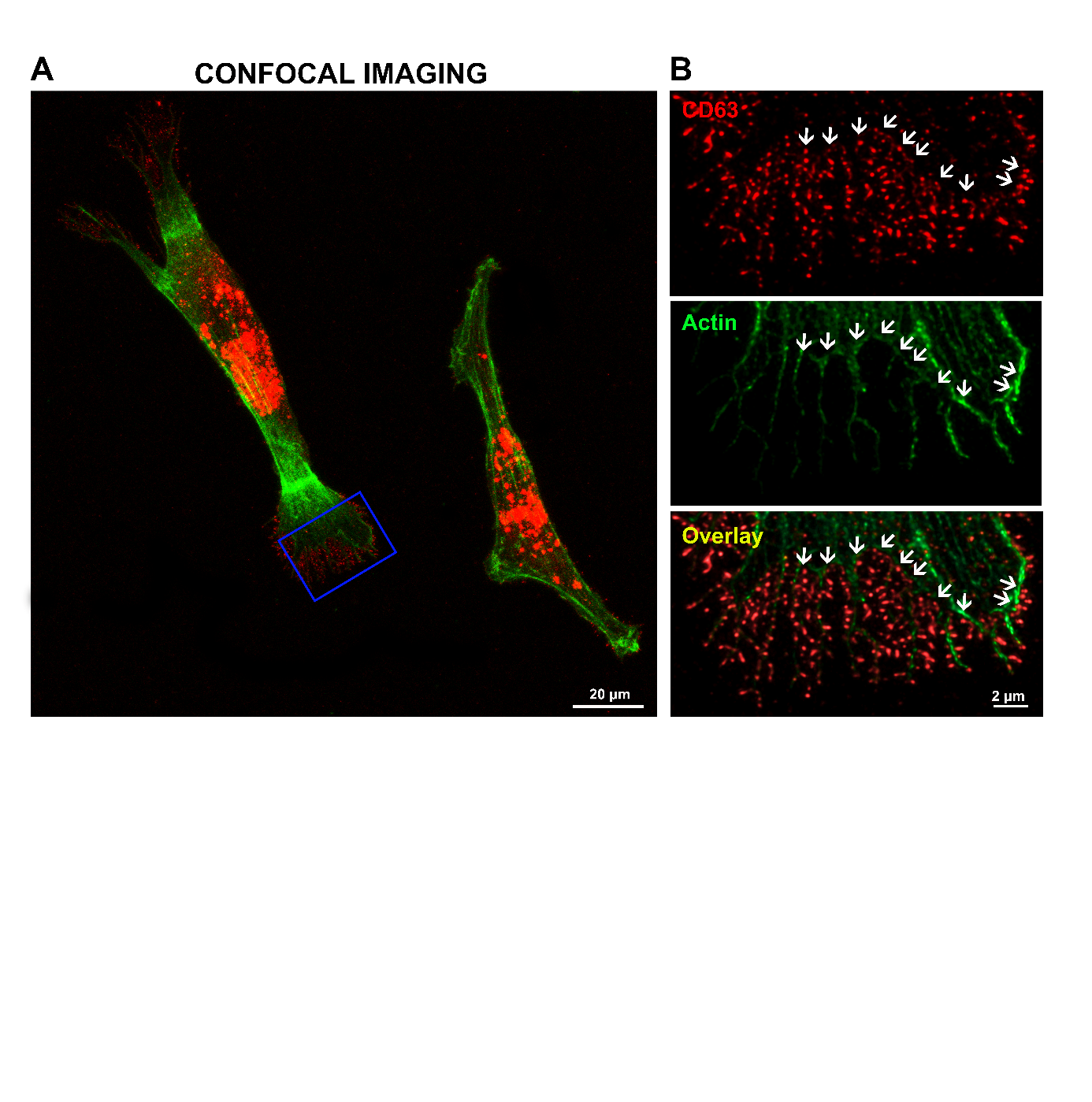


Supplementary Figure 3 alt text: Confocal image of glioma cells showing the live probe actin (green) and CD63 (red) staining. A magnified inset reveals the anchoring of actin filaments and the secreted exosomes in the extracellular space.

Supplementary Figure 3 – A) Representative deconvolved confocal image of OPBG-GBM001 cells stained with a specific permeant probe for F-actin filaments (SiR-Actin probe, pseudo-colored in green), post-fixed with paraformaldehyde and labeled with anti-CD63 antibody revealed by AlexaFluor 488-conjugated secondary antibody (pseudo-colored in red). B) Higher magnifications of the blue inset showing the cell periphery where the polymerizing actin filaments are anchored (arrows) and in the extracellular space with the secreted exosomes.

Supplementary Table 1

|  | **CD63-STAR Red Signal** | | | |
| --- | --- | --- | --- | --- |
|  | **Confocal** | **STED** | **dSTED** | **dSTED + FFT** |
| **mean intensity ± SD** | 235.65 ± 13.03 | 252.56 ± 6.16 | 253.30 ± 4.63 | 254.30 ± 3.48 |
| **mean intensity/SD (S/N)** | 18.09 | 41 | 54.71 | 73.07 |
| **mean intensity/mean bkg (S/B)** | 10.45 | 171.81 | 186.25 | 353.19 |

Supplementary Table 1 – Comparison between confocal and STED microscopy techniques, and mathematical processes of deconvolution of STED (dSTED) images carried out by Huygens software, followed or not by FFT filtering (dSTED+FFT). Signal-to-noise (S/N) and signal to background (S/B) measurements were performed in images of fixed and unpermeabilized cells stained with STAR Red-conjugated to CD63 antibody using ImageJ software.
